# Adaptive Switching Strategy of an Aerial Drone’s GNSS Antennas with Metallic Shielding for GNSS Anti-Jamming

**DOI:** 10.3390/s25185778

**Published:** 2025-09-16

**Authors:** Seojin Bang, Jonghoek Kim

**Affiliations:** System Engineering Department, Sejong University, Seoul 05006, Republic of Korea

**Keywords:** aerial drone, global navigation satellite system, anti-jamming method, metallic shielding structure, antenna selection method

## Abstract

This paper presents a simple and cost-effective anti-jamming method for global navigation satellite system (GNSS) antennas of an aerial drone. The drone has three GNSS antennas, such that two antennas exist at distinct heights inside a metallic shielding structure, and one antenna exists outside the shielding structure. To overcome intentional jamming signals from low elevation angles, we designed an autonomous antenna selection method. In the proposed antenna selection method, the most suitable GNSS antenna is dynamically selected to ensure stable satellite signal reception, while blocking the jamming signals from low elevation angles. Experiments were conducted to validate the proposed approach.

## 1. Introduction

Nowadays, the global navigation satellite system (GNSS) is important in many areas, such as the global location of aerial drones. However, GNSS signals are susceptible to jamming signals, which can hinder the ability of the aerial drone to navigate effectively. To address this issue, wire-guided aerial drones are often used to avoid jamming attacks from ground-based adversaries. However, wire-guided drones face limitations, including a limited operational range (determined by the length of the tether) and the risk of losing control if the tether is severed.

This paper proposes a wirelessly guided drone system that overcomes these limitations by allowing the aerial drone to be controlled over longer distances without the risk of wire disconnection. Furthermore, the proposed system is designed to avoid jamming attacks from ground-based sources, ensuring a more reliable GNSS signal reception and improved performance.

This paper presents a simple and cost-effective anti-jamming method for GNSS antennas of an aerial drone. The drone has three GNSS antennas, such that two antennas exist at distinct heights inside a metallic shielding structure, and one antenna exists outside the shielding structure. To overcome intentional jamming signals coming from low elevation angles, we designed an autonomous antenna selection method using a metallic shielding structure. In the proposed antenna selection method, the most suitable GNSS antenna is dynamically selected to ensure stable satellite signal reception, while blocking the jamming signals from low elevation angles.

A variety of studies have investigated GNSS anti-jamming techniques [1]. One approach employs a static conic metallic structure around the GNSS antenna to block low-elevation jamming signals [2], demonstrating the effectiveness of such metallic shielding. Backward-compatible single-antenna designs for GNSS spoof detection and anti-jamming in aviation applications have been proposed [3]. Spherical-cap adaptations of planar seven-element controlled reception pattern antenna arrays have also been explored to mitigate interference from low-horizon signals [4]. Polarization diversity has been leveraged in anti-jamming strategies as well. For example, space–time-polarization-domain adaptive processing (STPAPS) was introduced for single-element dual-polarized antennas [5], and adaptive beamforming algorithms using dual-polarized GNSS arrays have been developed to handle interference with various polarizations [6,7]. Antenna-based techniques for spoofing detection and mitigation, which utilize signal polarization to differentiate GNSS signals from interference and spoofing, have been described in [8]. This approach is capable of mitigating any linearly polarized interference. In this work [9], both horizontal and vertical choke-ring antennas are commonly used in geodetic-grade GNSS applications to mitigate low-elevation multipath signals. These antennas reflect low-elevation signals within their concentric groove structures, inducing path differences that lead to phase cancellation, thereby effectively attenuating unwanted multipath components while preserving high-elevation signals. In contrast, our approach combines a lightweight metallic shielding structure with an adaptive antenna selection algorithm, offering a simple and drone-compatible solution to mitigate low-elevation jamming without complex hardware or software modifications.

**Figure 1:**
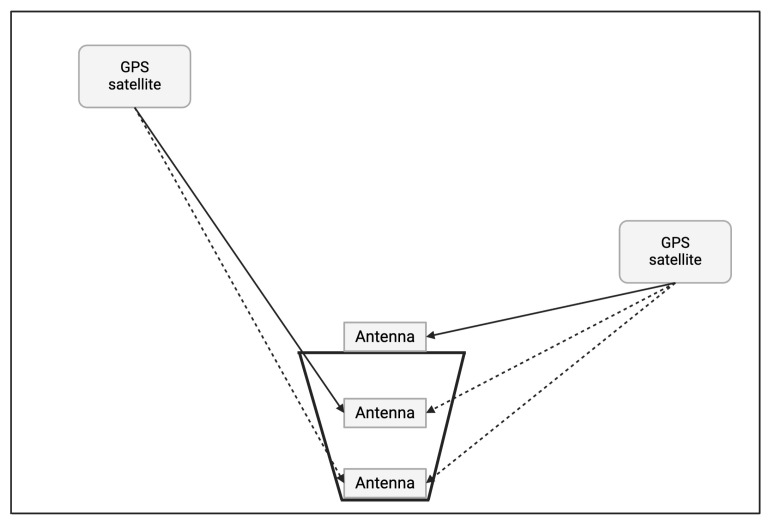
Conceptual illustration of how the metal shielding structure affects satellite signals at various elevation angles. Solid arrows indicate direct signals that reach the antenna, while dashed arrows denote signals blocked or attenuated by the shielding.

To overcome intentional jamming signals coming from low elevation angles, we build three antennas with a metal shield. We propose a simple antenna selection method using a metal shield. Figure 1 presents a conceptual illustration of how the metal shielding structure affects satellite signals at various elevation angles; as shown, the metallic shield around the antenna acts as a filter that blocks signals arriving from low-elevation angles. In other words, any jamming signals from ground-based sources (near the horizon) are naturally attenuated or excluded by the shield, while higher-elevation GNSS signals from satellites can still reach the antenna. In our approach, we deploy multiple GNSS antenna modules at different heights inside the shield and dynamically select the most suitable antenna in real time.

The upper (shallower) module is less shielded and can receive more satellites under benign conditions, whereas the lower (deeper) module is more shielded and filters out low-angle signals when interference is present. This height-based selection strategy ensures that the system always maintains reception from at least four satellites (the minimum for a valid GNSS fix) while mitigating interference from low-angle jamming sources. Essentially, the shield combined with adaptive module selection provides an effective elevation-based filter for jamming, without the need for sophisticated electronics or signal processing.

Real-world experiments were conducted to validate both the filtering effect of the shielding structure and the performance of the adaptive selection method. Although our experiments did not incorporate any intentional jamming signals, from the perspective of the GPS antenna—which cannot inherently distinguish between a low-elevation satellite signal and a jamming signal—we treated low-elevation signals as interference. This approach is based on the practical fact that signals arriving near the horizon degrade positioning accuracy in the same way as jamming signals originating from ground-based sources. Consequently, any signals coming from low angles can be blocked by the shield.

The remainder of this paper is organized as follows. Section 2 describes the antenna selection method and the system model. Section 3 presents experiments for the proposed antenna selection method. Finally, Section 4 concludes the paper.

## 2. System Model and the Antenna Selection Method

For regulatory and safety reasons, our evaluation is conducted without emitting artificial jamming signals. Unless intentionally carried by an airborne platform, GNSS jammers are deployed almost exclusively at ground level; their energy therefore reaches a receiver only through low-elevation paths (typically < 20°). Our shield is designed to preferentially attenuate this sector, and a passive shield’s insertion loss depends solely on incidence angle—not on the strength, modulation, or origin of the signal. Consequently, the attenuation measured on weak GPS satellites below 20° applies one-to-one to any strong interference arriving along the same geometry, providing a direct indicator of how effectively the shield would suppress a ground-based jammer. The RF front-end—comprising the low-noise amplifier, band-pass filters, mixers, and automatic gain control (AGC)—holds the total in-band power close to the broadband thermal-noise floor. Under normal satellite-only conditions, the input power stays near that floor, so the AGC remains stationary. When a strong low-elevation continuous-wave (CW) jammer appears, however, it drives the in-band power above the floor; the AGC reacts by cutting gain, and the correlator C/N0 collapses [10]. Because this gain reduction can be triggered only by energy entering from the same low-elevation geometry characteristic of ground-based jammers, physically blocking that angular sector keeps the AGC within its optimal operating range. By demonstrating more than 20 dB-Hz of attenuation for signals arriving below 20° elevation in a jammer-free environment, we conservatively validate that the same hardware filter would reject comparable energy from an actual ground-level jammer. Therefore, this jammer-free test provides a reliable proof of concept.

For real-time monitoring and data collection, our experiments employ two key NMEA sentences:

### 2.1. Parameters

GPGSV (GNSS Satellites in View): The GPGSV message provides the PRN number, elevation, azimuth, and C/N0 for each satellite. From these data, per-satellite signal strength can be extracted, and the fraction of visible satellites below a specified elevation threshold (e.g., 20°) can be computed. These values are useful for characterizing the geometry of the satellite and evaluating signal quality at different elevation angles.

GPGGA (GNSS Fix Data): The GPGGA message provides essential fix information, including UTC time, latitude and its hemisphere (N/S), longitude and its hemisphere (E/W), GNSS quality indicator (fix status: 0 = invalid; 1 = standard fix) and the number of satellites used in the fix. To evaluate the validity of the receiver’s fix and monitor signal conditions, we solely use the fix status and the number of satellites used in the solution.

Fix Status: Indicates whether the GNSS antenna has successfully acquired a valid and reliable position fix.

Satellite Count: Represents the number of satellites currently being tracked and used by the GNSS module for position computation.

Elevation: The angle between the satellite and the local horizon, which influences signal quality due to atmospheric attenuation and obstructions at low angles.

C/N0 (Carrier-to-Noise density ratio): Quantifies the strength of the received satellite carrier signal relative to the noise power spectral density, expressed in dB-Hz, serving as a key indicator of GNSS signal quality.

### 2.2. Antenna Module Selection Method

The drone has three GNSS antennas, such that two antennas exist at distinct heights inside the metallic shielding structure, and one antenna exists outside the shielding structure. To overcome intentional jamming signals coming from low elevation angles, we designed an autonomous antenna selection method.

The antenna which exists outside the shielding structure has the largest signal strength. Then, the antenna at the shallow position inside the shielding structure has a medium signal strength. The antenna at the deepest position inside the shielding structure has the lowest signal strength. As the currently used module has increased signal strength, the antenna typically captures signals from more satellites. We say that an antenna signal strength (SS) is large, as it has large signal strength.

We outline the procedure for selecting the optimal GNSS antenna module from several modules positioned at different positions. To ensure a valid 2D fix, at least three satellites must be tracked, as defined by the NMEA GPGGA standard (Fix Status = 1). An upper bound is introduced to represent the satellite count beyond which the antenna is likely to receive low-elevation signals, which may include interference. The algorithm leverages this threshold to determine when to switch modules in order to maintain optimal signal quality.

The selection process is initiated by choosing the module at the lowest signal strength, to avoid excessive exposure to low-elevation interference. If the current module fix status is 0 (no valid fix), the controller switches to the next larger SS module to seek a higher satellite count. Conversely, if the fix status is 1 but the number of satellites used in the fix exceeds a predefined upper bound, the algorithm interprets this as the antenna receiving low-elevation signals—potentially including interference—and switches to the next smaller SS module. Otherwise—meaning the fix is valid and the satellite count is within the acceptable range—the system continues using the current module.

At each cycle, the Arduino reads FixStatus and SatelliteCount from the currently active GNSS module and applies the following three rules:

- FixStatus = 0 (no position fix) → switch to the next larger SS module.
- FixStatus = 1 and SatelliteCount > upper bound → switch to the next smaller SS module.
- Otherwise, → stay on the current module.

This dynamic switching mechanism ensures that the GNSS antenna remains in an optimal reception state, balancing the need for adequate satellite visibility with the avoidance of low-elevation interference. The upper bound should be tuned according to system requirements and environmental conditions. This selection process runs continuously in a real-time monitoring loop, ensuring that the GNSS antenna maintains a stable and interference-resilient connection. Unlike existing interference-mitigation approaches such as FFT excision filtering implemented on FPGA [11] (O(n log n)) or adaptive beamforming algorithms [12] (O(n^2^)), which are computationally intensive and often unsuitable for real-time processing on small aerial platforms, our proposed rule-based selection strategy operates in constant time O(1) with only ≈10–20 primitive operations per decision. This extremely lightweight complexity enables real-time execution even on low-power microcontrollers, making it well-suited for embedded drone applications.

**Figure 2:**
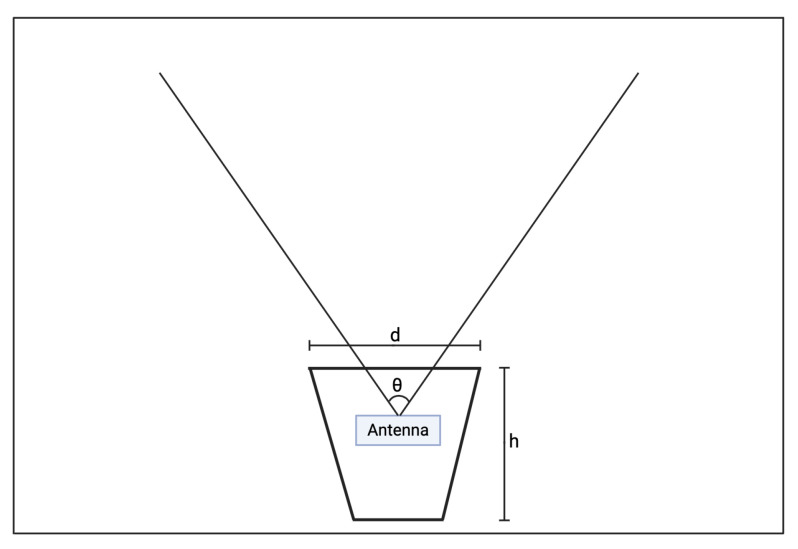
Schematic of the shielding structure, showing its diameter (d), height (h), and opening angle (θ) that defines the minimum beamwidth for mitigating low-elevation signals.

Figure 2 shows the schematic of the shielding structure, showing its diameter (d), height (h), and opening angle (θ) that defines the minimum beamwidth for mitigating low-elevation signals. Table 1 shows the hardware information of the shielding structure.

A cylindrical shielding structure was used to selectively attenuate low-elevation GNSS signals. As illustrated in Figure 2, the configuration includes two GNSS modules placed at different depths within the shielding body and a third module positioned externally to serve as the largest SS module in the switching algorithm. One internal module is installed at a height corresponding to an 84° beamwidth based on line-of-sight geometry, which is theoretically sufficient to ensure reception from at least four satellites, assuming an even spatial distribution of 32 satellites in the sky [13]. This design ensures angular separation between modules, making it possible to compare performance under different signal conditions.

## 3. Experiments

### 3.1. Experiment 1: Verification of the Filtering Effect of the Shield

This experiment validates the shield’s built-in low-elevation filter by logging GNSS C/N0 from three antennas: an external reference outside the shield, a “shallow” antenna mounted 3 cm above the shield base, and a “deep” antenna at the shield base. Comparing their C/N0 versus elevation reveals (i) how strongly the shield alone suppresses signals below 20°, and (ii) the extra attenuation contributed by antenna depth, while confirming that high-elevation reception remains largely unaffected.

#### 3.1.1. Experimental Setup

**Shielding Structure**

- Geometry: Cylinder, 4.5 cm diameter × 5 cm height.
- Materials: Exterior wrapped in aluminum foil to block low-elevation signals; interior lined with non-conductive black tape to minimize internal reflections.
- Aperture: Top opening restricted to 1.8 cm, admitting predominantly high-elevation rays.

**GNSS Modules**

**Figure 3:**
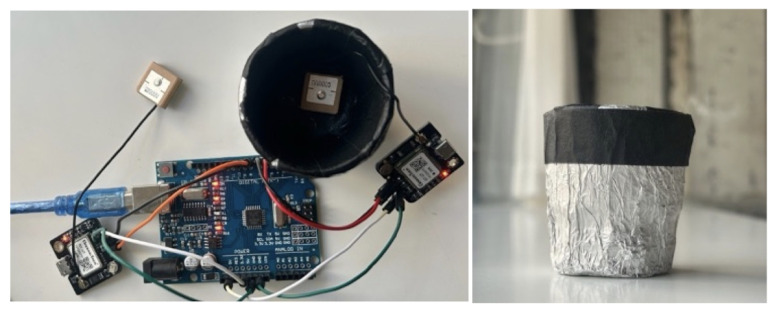
Experimental setup showing the Arduino-based data logger with three GT-U7 modules and the metallic shield (4.5 cm diameter × 5 cm height, exterior wrapped in aluminum foil, interior lined with black non-conductive tape). The top aperture (1.8 cm) is not visible in this view.

Figure 3 shows the experimental arrangement: an Arduino Uno-based data logger (Arduino, Somerville, MA, USA) with three GT-U7 GPS modules (Geekstory) and the cylindrical metal shield (4.5 cm × 5 cm; aluminum exterior, non-conductive lining; 1.8 cm top aperture, not visible here).

**Figure 4:**
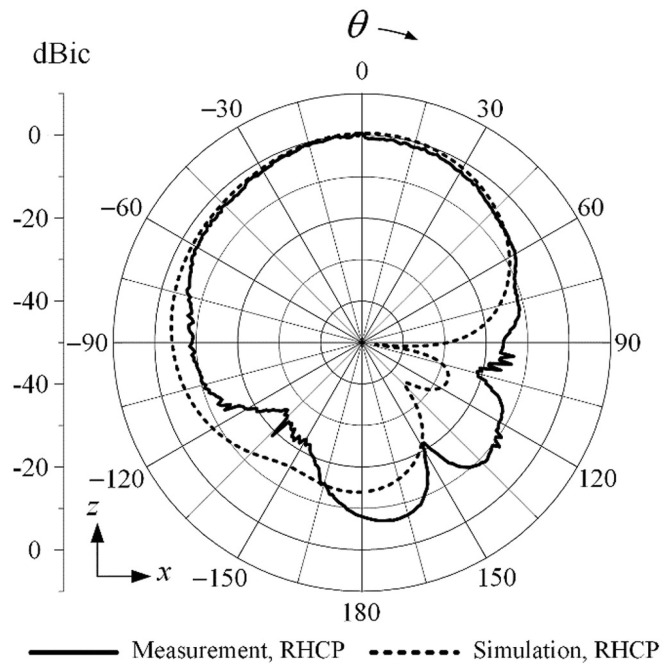
Two-dimensional radiation pattern at GNSS L1 (1.575 GHz), showing RHCP gain distribution in the zx-plane. Adapted from [14].

Figure 4 shows the 2D radiation pattern at GNSS L1 (1.575 GHz), showing RHCP gain distribution in the zx-plane. This figure is adapted from [14]. GNSS receiver locations are presented in Table 2.

- Configuration: Three GT-U7 modules (L1 band, up to 50 channels) were used in the experiment.
- Active Antennas: Gain ≥ 15 dB, noise figure ≤ 1.5 dB to offset cable loss and ensure high sensitivity.
- Polarization: GNSS L1 signals are RHCP, so the in-shield antennas do not require precise zenith pointing; this eliminates orientation as a confounding factor for C/N0 comparison [14].

Data logging

Each GT-U7 receiver transmits a full NMEA sentence set at 1 Hz, but a 3-to-1 UART multiplexer connects to only one module at a time. The connection dwells for 5 s on the external antenna (0–5 s), then switches to the shallow antenna (5–10 s), and finally to the deep antenna (10–15 s), completing a 15 s cycle. Consequently, every module is observed for a single 5 s window once every 15 s. During its active window, the logger stores the five consecutive GPGSV frames and retains the maximum C/N0 value; this choice minimizes errors due to fading or transient interference while capturing each antenna’s peak sensitivity.

Measurement protocol Data were collected in an open field offering an unobstructed 360° sky view, ensuring direct line of sight to all visible satellites. Following the GT-U7 specification, each receiver was cold-started and allowed 30 s to acquire a stable GNSS fix before the 30 min logging session began.

#### 3.1.2. Measurement Summary

**Figure 5:**
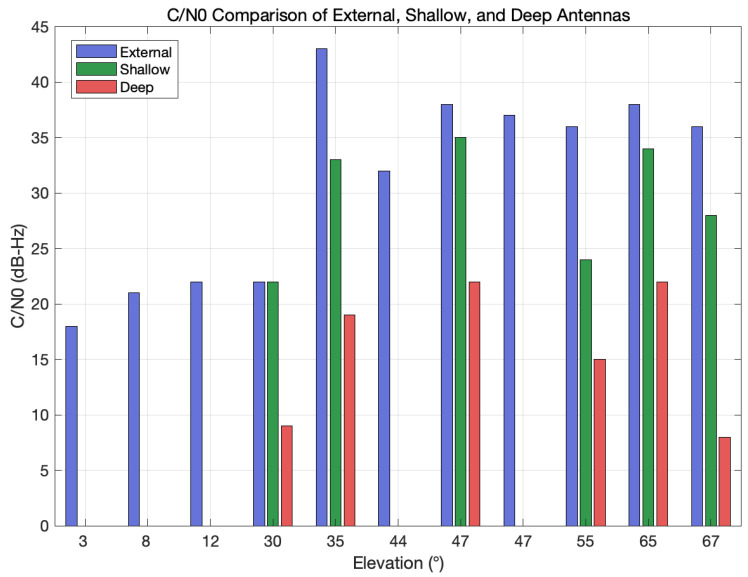
Carrier-to-noise density ratio (C/N0) comparison for the external (blue), shallow (green), and deep (red) antennas as a function of satellite elevation angle, illustrating the shield’s near-horizon suppression and depth-controlled attenuation.

Figure 5 shows the carrier-to-noise density ratio (C/N0) comparison for the external (blue), shallow (green), and deep (red) antennas as a function of satellite elevation angle, illustrating the shield’s near-horizon suppression and depth-controlled attenuation.

First, the observations were binned into three elevation ranges: low (0°–20°), mid (20°–50°), and high (>50°). For each range, we retained the peak C/N0 as the representative value. Table 3 summarizes the measurement results for three elevation bands.

The key observations are as follows. The shield demonstrated near-horizon suppression by completely blanking all satellites at elevations of 20° or below: both in-shield antennas reported 0 dB-Hz, corresponding to at least a 20 dB-Hz reduction relative to the external reference. In the 20°–50° band, the shallow antenna achieved a maximum C/N0 of 18 dB-Hz, whereas the deep antenna’s maximum C/N0 was only 10 dB-Hz; the resulting 8 dB-Hz differential confirms that antenna depth can tune the filter’s aggressiveness. At elevations above 50°, the shallow antenna suffered only an 8 dB-Hz penalty—maintaining approximately 29 dB-Hz of link margin—while the deep antenna lost about 22 dB-Hz, reducing its maximum C/N0 to 15 dB-Hz, which is still adequate for tracking but offers a limited fade margin.

Compared to the metallic conical structure reported in [2], which reduced the horizontal-plane gain by 6.2 dB, our shielded in-antenna measurements indicate a reduction of roughly 20dB-Hz in C/N0 for low-elevation signals. While these values are expressed in different units and cannot be directly compared quantitatively, the results suggest that our shielding approach provides correspondingly stronger suppression of low-elevation interference.

These results verify that the shield alone is sufficient to eliminate low-elevation interference, and deeper antenna placement offers stronger suppression at the cost of reduced high-elevation C/N0. Consequently, a hybrid strategy that operates normally with the shallow antenna and switches to the deep antenna only under jamming conditions could provide an optimal balance between robustness and navigation availability.

### 3.2. Experiment 2: Antenna Module Selection Method

This experiment extends the findings of Experiment 1 by evaluating a real-time GNSS receiver selection algorithm that leverages the elevation-filtering properties of a metallic shield. Instead of manually comparing fixed antennas, we test whether an Arduino-based controller can maintain a valid GNSS fix by switching among three modules at different SS, based on live GPGGA data.

#### 3.2.1. Experimental Setup

Shielding Structure

We reuse the cylindrical shield from Experiment 1 (Ø 4.5 cm × 5 cm, 1.8 cm aperture) and deploy three GT-U7 modules: one at the base inside the shield (deep, 0 cm), one 3 cm above it (shallow), and a third module outside the shield (external). All three modules are candidates in the real-time selection algorithm.

Data Logging

All three GNSS modules output standard NMEA sentences at 1Hz. The Arduino logger filters both GPGSV and GPGGA messages and stores each record with the following fields: timestamp, PRN (satellite ID), elevation, C/N0, FixStatus, and SatelliteCount. Here, FixStatus is taken from GPGGA (1 = valid position fix, 0 = no fix), while SatelliteCount is the number of satellites contributing to that fix. Collecting these aligned parameters enables time-synchronized analysis of how the module-switching algorithm responds to environmental changes and the shield’s attenuation effects.

Selection Algorithm Implementation

At each cycle, the Arduino reads FixStatus and SatelliteCount from the currently active GNSS module and applies three rules in Section 2.2. This rule set lets the controller climb upward toward modules with better sky view when a fix is lost and descend to deeper, better-shielded positions when plenty of satellites are available.

#### 3.2.2. Measurement Summary

Low-angle signal ratio is defined as the fraction of all received satellites whose elevation angles are ≤20°, which we treat as the proxy for potential ground-level jamming signals.

**Figure 6:**
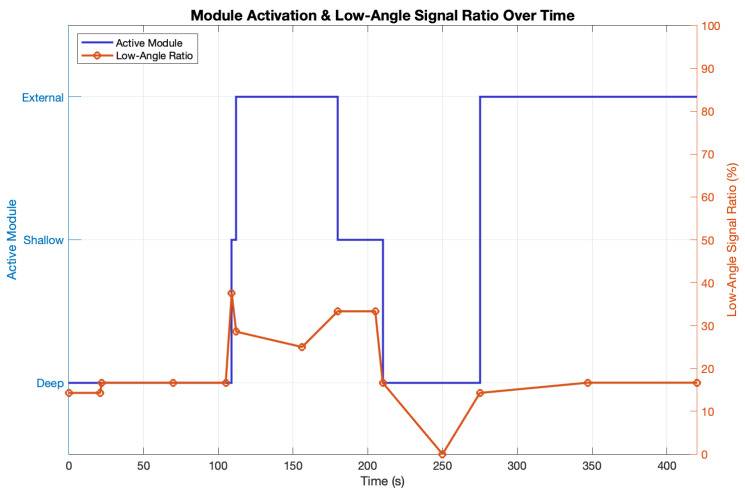
Time series of GNSS module switching behavior (left axis) and low-elevation signal ratio (right axis) during Experiment 2. The system dynamically selects the most appropriate receiver based on fix stability and satellite count thresholds. Low-angle signal surges correlate with module transitions from strong SS to deep antennas.

Figure 6 shows how the dynamic-selection algorithm works in concert with the shield’s elevation filter. During open-sky intervals, the controller stays on the deep module and the fraction of low-elevation (<20°) satellites is held below 30%—well under the 67% expected from pure geometry. When the platform moves indoors at about 105 s, SatCount falls and the position fix is lost, so the algorithm climbs to the shallow and then external modules until the fix is regained. In intermediate conditions (e.g., near a window at 160 s) SatCount exceeds the upper threshold, prompting a descent to the shallow module; re-entering open sky around 205 s drives a further descent back to the deep module as low-elevation signals again dominate. A final underground segment (350 s) produces severe blockage, repeated upward switches, and ultimately loss of fix on all modules. Overall, the hybrid hardware–software scheme suppresses low-elevation signal power whenever conditions allow, yet automatically relaxes the filtering to preserve navigation availability when the signal environment deteriorates.

**Figure 7:**
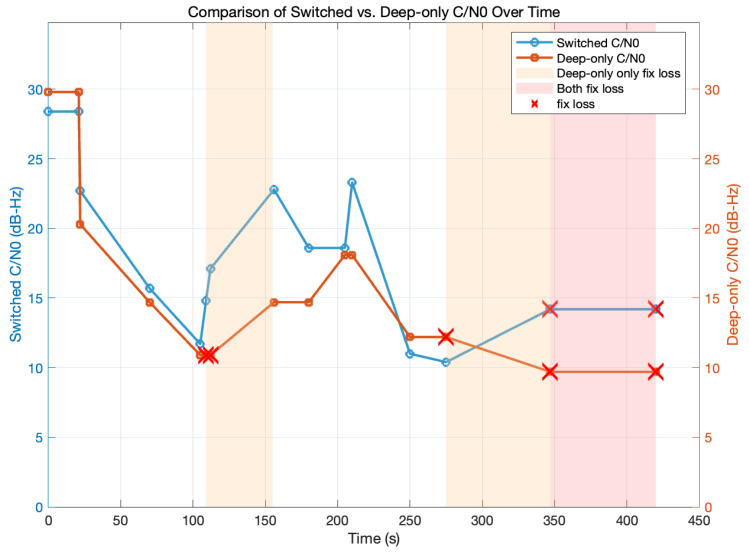
Time-series comparison of switched (blue line) versus deep-only (orange line) maximum C/N0. Shaded bands denote periods when only the deep-only configuration loses fix (light orange) and when both configurations lose fix (red), and red “×” markers indicate individual fix-loss events (FixStatus = 0).

Figure 7 compares the maximum C/N0 of the dynamic-switch scheme (blue) with the deep-only baseline (orange). In open-sky conditions (0–70 s), both modes held 23–28 dB-Hz C/N0 and never lost lock. Once partial or deep indoor shadowing occurred (70–155 s), the deep-only antenna dropped below the 12 dB-Hz threshold and repeatedly lost its fix, whereas the dynamic-switch algorithm moved to the stronger SS antenna, restored C/N0 to 17–23 dB-Hz, and kept the fix. Under extreme attenuation (347–420 s), neither scheme could sustain more than 14 dB-Hz, so both eventually lost lock. Overall, dynamic switching preserved a valid fix for 83% of the run, versus only 54% for the deep-only configuration.

### 3.3. Experiment Analysis

The results of Experiment 2 show that a geometry-aware, elevation-selective strategy is viable in practice: a structurally shielded antenna suppresses low-angle interference, while real-time fix-driven switching preserves navigation availability whenever signal conditions deteriorate. Together, the hardware–software hybrid improves GNSS robustness under partial blockage and simulated jamming, fully consistent with the shielding behavior observed in Experiment 1. Although antenna switching at 1 Hz may cause short-term phase tracking interruptions, the effect remained within the receiver’s tolerance and did not compromise navigation availability in our experiments, since the drone’s relatively low flight speed ensures that sub-second interruptions do not accumulate into significant positioning errors.

## 4. Conclusions

We address a simple and cost-effective anti-jamming method for GNSS antennas of an aerial drone. The drone has three GNSS antennas, such that two antennas exist at distinct heights inside the metallic shielding structure, and one antenna exists outside the shielding structure. To overcome intentional jamming signals coming from low elevation angles, we addressed an autonomous antenna selection method. In the proposed antenna selection method, the most suitable GNSS antenna is dynamically selected to ensure stable satellite signal reception, while blocking the jamming signals from low elevation angles. Real-world experiments were conducted to validate the proposed approach.

## Figures and Tables

**Table 1:** Hardware information of the shielding structure.

d (Diameter of shielding structure)	4.5 cm
h (Height of shielding structure)	5 cm
θ (threshold for shallow module placement to mitigate jamming)	84°

**Table 2:** GNSS receiver locations.

Channel	Location Description
External	Outside the shield (reference antenna)
Shallow	3 cm above the shield base (2 cm headroom)
Deep	On the shield base (0 cm; maximum depth)

**Table 3:** Average C/N0 and attenuation by elevation band.

Elevation Band	Mean Peak C/N0 (dB-Hz)			Attenuation ^†^
	External	Shallow	Deep	Shallow/Deep
Low (0–20°)	20.3	**0.0**	**0.0**	−20.3/−20.3
Mid (20–50°)	34.4	18.0	10.0	−16.4/−24.4
High (>50°)	36.7	28.7	15.0	−8.0/−21.7

^†^ Attenuation is calculated as External—(Shallow or Deep).

## Data Availability

The original contributions presented in this study are included in the article. Further inquiries can be directed to the corresponding author.
